# Slippery liquid infused fluoropolymer coating for central lines to reduce catheter associated clotting and infections

**DOI:** 10.1038/s41598-020-71711-6

**Published:** 2020-09-11

**Authors:** Saibal Bandyopadhyay, Andrew Jones, Andrew McLean, Matthew Sterner, Carolyn Robbins, Matthew Cunningham, Mark Walters, Kiran Doddapaneni, Isaac Keitel, Colin Gallagher

**Affiliations:** 1 FreeFlow Medical Devices LLC, Lancaster, PA USA; 2 American Preclinical Services LLC, Minneapolis, MN USA; 3 Shared Material Instrumentation Facility, Duke University, Durham, NC USA

**Keywords:** Bacterial infection, Biomaterials, Biomedical materials, Implants, Disease prevention

## Abstract

Thrombosis and infections are two grave, interrelated problems associated with the use of central venous catheters (CVL). Currently used antibiotic coated CVL has limited clinical success in resisting blood stream infection and may increase the risk of emerging antibiotic resistant strains. We report an antibiotic-free, fluoropolymer-immobilized, liquid perfluorocarbon-coated peripherally inserted central catheter (PICC) line and its effectiveness in reducing catheter associated thrombosis and pathogen colonization, as an alternative to antibiotic coated CVL. Commercially available polyurethane PICC catheter was modified by a three-step lamination process, with thin fluoropolymer layers to yield fluoropolymer–polyurethane–fluoropolymer composite structure before applying the liquid perfluorocarbon (LP). This high throughput process of modifying commercial PICC catheters with fluoropolymer is quicker, safer and shows higher thromboresistance than fluorinated, omniphobic catheter surfaces, produced by previously reported self-assembled monolayer deposition techniques. The LP immobilized on the fluoropolymer is highly durable in physiological flow conditions for over 60 days and continue to resist Staphylococcus colonization.

## Introduction

Indwelling central venous catheters (CVL) are widely used in critically ill patients for long-term vascular access to administer nutrition, dialysis, diagnostics, lifesaving antibiotics and chemotherapy, accounting for nearly 8% of the US hospital population at any given time^1^. Although widely used, CVL implantation causes significant morbidity and mortality every year^2,3^ due to its associated complications such as biofilm formation, central line-associated blood stream infections^2,4,5^ (CLABSIs), fibrin sheath development, and thrombosis. For CVLs, infection and fibrin sheath formations are two closely related problems^6,7^. Triggered by the vessel injury during catheter implantation, fibrinogen, albumin, lipoproteins and coagulation factors begin to deposit on the catheter surface within 24 h, thus developing the fibrin sheath, which covers the entire catheter within a few days. This fibrin sheath is primarily responsible for the late stage catheter dysfunction, which usually occurs about three months after the catheter placement. Other than catheter dysfunction, fibrin sheath formation also has a pathophysiological relation with biofilm growth, where bacteria colonies, protected by an extracellular polymeric matrix also known as glycocalyx, show 1,000 times high resistance to systemic antibiotics than their planktonic counterpart^8,9^. CLABSI pathogens enter the bloodstream through both extraluminal and inner luminal wall of implanted catheter. Seeding of bacterial pathogens from biofilm also induces CLABSI. The 250,000–500,000 CLABSI incidences per year in the US create a significant burden on the healthcare system, with expenses over a $1 billion per year ($16,000 to $32,000/patient)^10^ and mortality rate at 10–25%^11^.

The US Centers for Disease Control and Prevention (CDC) released a checklist for hospitals and physicians, including proper insertion methods as well as central line handling and maintenance, to reduce the number of CLABSI^12^ occurrences. Although this successfully reduced the rate of CLABSI by ~ 50%, no subsequent efforts to improve the outcomes have been successful in a clinically meaningful way^13^. To further reduce CLABSI and catheter dysfunction, coating CVL surface with anti-microbial, antibiotic and anticoagulant materials has been a popular approach. Catheters coated with silver^14^, bismuth, chlorhexidine and silver sulfadiazine^15^, rifampicine and minocycline^16^ have marginally reduced CLABSI rate in clinical setting but are still not widely recommended, except in high risk patient populations e.g. surgical patients with cancer or infection, immunocompromised, adult burn patients or infants^17^. Bio-passive surface modification to target both thrombosis and infection has emerged as a more successful approach for medical devices and implants. Fluoro-passivation of medical implants is a bio-passive surface modification approach where the medical device surface is coated with fluoropolymer or modified with fluorinated additives to increase their biocompatibility and thereby reduce inflammatory response and thrombogenicity. Several notable commercialized examples in this category are poly(bis(trifluoroethoxy)phosphazene) coated COBRA PzF^18^ and poly(vinylidene fluoride-co-hexafluoropropylene) coated XIENCE Sierra coronary stents^19^; AngioDynamics BioFlo (endexo) PICC catheter and CerebroFlo (endexo) extraventricular drain catheter incorporate A-B-A block copolymer in TPU^20^ or silicone rubber as additive. More recently, liquid perfluorocarbon (LP) coatings have emerged as a novel class of slippery omniphobic coatings which was originally produced by infusing liquid perfluorocarbon e.g. perfluorodecalin into a porous fluorinated surface^21^ which can resist bacterial colonization^22^. Since most medical device surfaces are smooth, subsequently, a more versatile two-step approach evolved which presumably added a fluorinated self-assembled monolayer (F-SAM) on non-porous medical device surfaces via solution^23^ or vapor phase^24^ surface grafting of fluorinated silane (F-silane) molecules (e.g. tridecafluoro-1,1,2,2-tetrahydrooctyl trichlorosilane). An LP layer was then applied on the F-SAM layer to produce LP/F-SAM, a slippery omniphobic coating which showed outstanding dual performance: (1) higher thromboresistance than heparin coated surfaces; and (2) more effective resistance to bacterial attachment and colonization than PEG^25^ and albumin^26^ coated surfaces. The durability of the LP/F-SAM coating within the inner lumen of a catheter against physiological shear stress was also demonstrated ex vivo^23^. Dual efficacy of this slippery omniphobic coating makes it a promising candidate to eliminate fibrin sheath formation and reduce the occurrences of CLABSI in CVL applications.

The biggest downside of the F-SAM grafting technique is that both solution and vapor phase F-silane grafting processes require pre-activation of the device surface with oxygen plasma and a post-grafting thermal condensation process, making the overall coating process lengthy (20 h per batch) and thus, cost inefficient for inexpensive medical catheters. These processes also require a surface purification step where the hydrolyzed yet unconjugated F-silane monomers and oligomers produced by homopolymerization process are rinsed off, preferably using a perfluorinated solvent in which the F-silane is highly soluble. To overcome these practical limitations of the chemical grafting process, we engineered a mechanical process where a 25–50-micron thick fluoropolymer layer is laminated on both inner lumen and the outer surface of commercially available peripherally inserted central catheters (PICC), a common type of CVL with high rates of CLABSI occurances^27^. This mechanical modification is compatible with the one step catheter extrusion process currently used for its mass production. The fluoropolymer laminated PICC is then dipped into the LP to obtain the PICC coated with a fluoropolymer immobilized liquid perfluorocarbon (FILP). Herein, we compared the surface topography and elemental composition of FILP coated PICC catheter (FILP PICC) with the LP/F-SAM coated PICC catheter (LP/F-SAM PICC) and also evaluated their thromboresistance in a comparative study with other commercially available PICC catheters: BARD PowerPICC (BARD PICC) and AngioDynamics BioFlo PICC (BioFlo PICC), which also use a fluoro-passivation approach. We have also shown that the FILP coating on PICC lines is durable under physiologically relevant flow conditions for over 60 days and can continue to resist the colonization of bacterial pathogens *Staphylococcus epidermidis* (*S. epi*) and *Staphylococcus aureus* (*S. aureus*) which are known to cause CLABSI in hospital settings.

## Results

### Selection of fluoropolymer for FILP coating

First, we set out to select suitable fluoropolymers which can support a layer of LP coating against shear force and the resulting surface would slide off of a 5 µL water droplet when the surface is tilted at 5-degree angle, a characteristic property well known for slippery omniphobic coatings. Commercially available 125-micron thick films of different fluoropolymers e.g. polytetrafluoroethylene (PTFE), fluorinated ethylene propylene (FEP), perfluoroalkoxy alkane (PFA), polyvinylidene fluoride (PVDF) and ethylene tetrafluoro ethylene (ETFE) were attached adjacent to each other on a 20 × 25 cm aluminum plate, for direct comparison. A Pebax film of comparable thickness (negative control) and an F-SAM deposited Pebax film (positive control, shows slippery omniphobic properties) produced by the previously reported CVD technique^24^ were also attached to the same aluminum plate. All polymer films were lubricated with perfluorotributyl amine (LP) using a paint brush, and the excess LP was removed by vertically submerging the plate in a water bath. All LP coated films were tested for the slippery hydrophobic property by placing five water droplets (5 µL each) per film using a multichannel pipette while the plate was secured at a 5-degree angle. We defined failure when more than two droplets failed to slide off the surface. Only, LP immobilized on PTFE, FEP, PFA and F-SAM deposited Pebax (LP/F-SAM) films allowed all water droplets to slide off, whereas PVDF, ETFE and Pebax retained all five water droplets. In the next round of testing, we selectively compared PTFE, FEP, PFA and F-SAM deposited Pebax films after they were freshly lubricated with perfluorotributyl amine (LP). This time the plate was dipped in water bath consecutively in sets of 5, 10, 15, 20, 25 and 30 dippings, and after each set of dipping the water sliding ability was tested in similar manner, i.e., 5µL water droplets were placed on the films which were tilted at 5-degree angle. After dipping for five times, only PTFE, FEP and PFA allowed all five droplets to slide off, whereas more than three droplets failed to slide off the F-SAM deposited Pebax surface. The results are summarized in Fig. S-1 (supporting information). We repeated the experiment with heparinized sheep blood instead of water droplets and the blood droplet (20 µL) also slid off the surface at 5-degree slide angle (Video S-1). We also confirmed that PTFE, FEP or PFA does not show this low slide property with blood or water droplets in the absence of the LP layer, or if we allow the LP layer to evaporate off these fluoropolymer surfaces by exposing them to air flow for 30 min before the sliding test. The LP immobilized on PTFE, FEP and PFA surfaces survived dipping up to 30 times, indicating the durability of the omniphobic coating, whereas the LP/F-SAM deposited surfaces failed to pass the slide test after five dipping in all three replicated tests.

### Slide angle and contact angle measurement

Hydrophobicity and slippery omniphobicity of the surfaces were evaluated by measuring water contact angles and slide angles on fluoropolymer surfaces, before and after the application of a LP layer. Three different LPs, perfluorodecalin, perfluorotributylamine, and perfluorotripentylamine of different viscosities and surface tensions (Table S-3, supporting information), were tested and the contact angle data is summarized in table S1 (supporting information). Static water contact angles on PFA, FEP, ETFE, and PVDF increased upon the addition of either of the three LP layers (Fig. S-2, supporting information) on the fluoropolymer surface. Perfluorodecalin showed an increase (~ 15°) in contact angle, when applied on both PFA and FEP surfaces. Contact angles on PTFE did not show any significant change upon the addition of either of the three LPs. In a sharp contrast, F-SAM deposited Pebax showed a reduction of more than 15° in contact angle upon the addition of either of the three LP layers. Sliding angle was measured as the minimum tilting angle required for a 5 µL water droplet to start sliding off the surface. Sliding angle expressed in logarithmic scale is summarized in Fig. S-3 (supporting information). The pure fluoropolymer surfaces showed > 45° sliding angle which dramatically decreased to 3°, 1° and 2.5° for PTFE, FEP and PFA, respectively, when these fluoropolymer surfaces were coated with the LP; but no significant change was observed on PVDF or ETFE surfaces upon the addition of any LP layer. A similar decrease in sliding angle was noted on F-SAM deposited Pebax surface as reported before^24^. Since perfluorotributylamine has the lowest surface tension, low volatility and showed a sliding angle comparable to perfluorodecalin which has the lowest sliding angle among all three LP tested, we decided to use it as the LP layer for the following experiments.

### Producing FILP PICC

**Figure 1:**
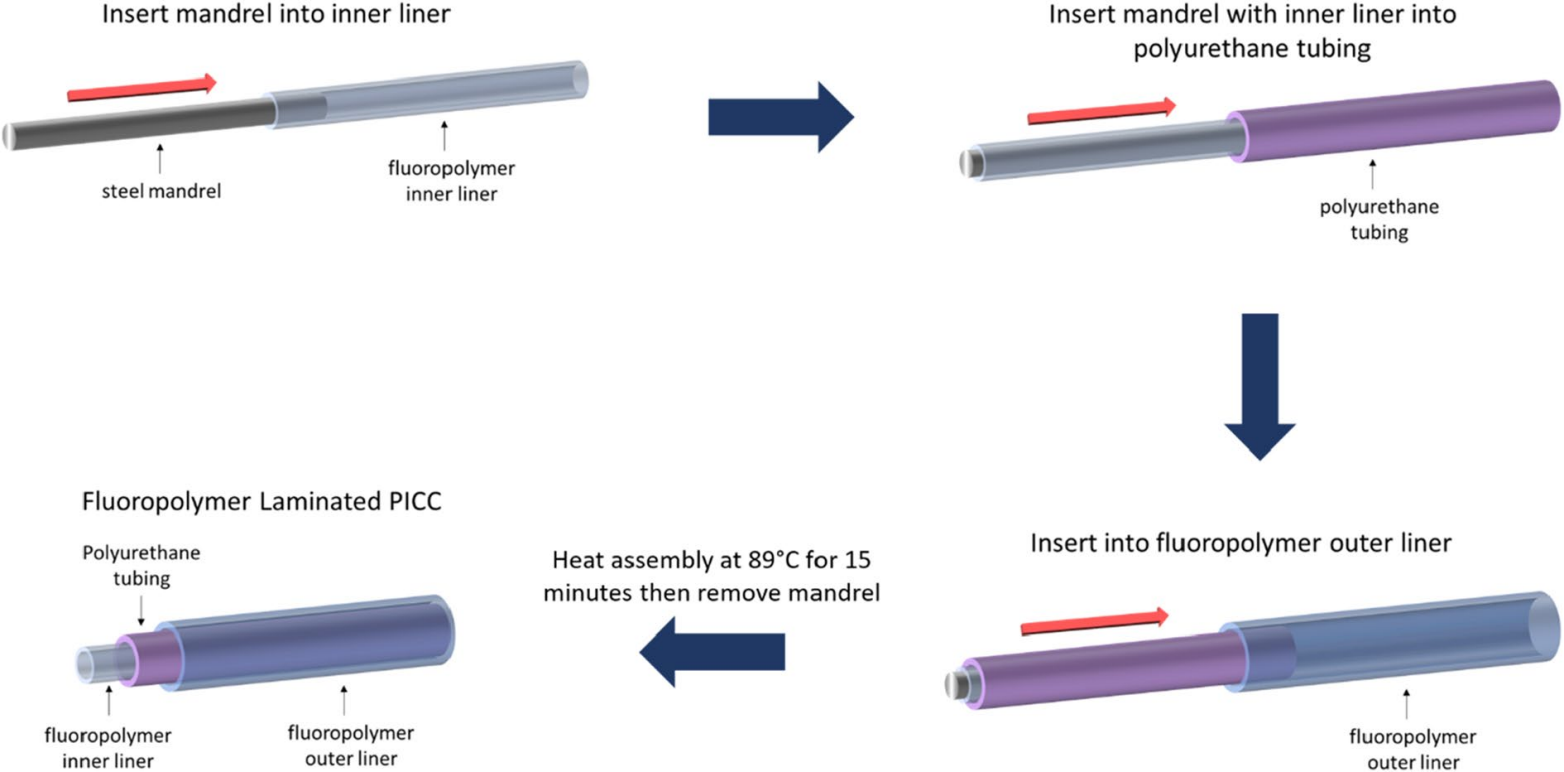
Fluoropolymer laminated single lumen PICC catheter manufacturing process to obtain the three-layer PTFE–TPU–FEP structure.

Perfluorinated plastics e.g. PTFE, FEP, PFA are hard^28^ and non-elastomeric materials, hence, PICC lines produced with pure fluoropolymers lack the mechanical flexibility required for use as an indwelling catheter. To maintain the flexibility of FILP coated PICC we decided to incorporate a thin layer of perfluoropolymer (PTFE, FEP or PFA) in both inner lumen and on the outer surface of the commercially available thermoplastic polyurethane (TPU) PICC, yielding a three-layer perfluoropolymer-TPU-perfluoropolymer composite structure. The process of modifying a BARD PICC with the fluoropolymer layers is shown in Fig. 1. For the inner liner, a commercially available PTFE sleeve was selected with a 25 ± 12 micron wall thickness and a 0.89 mm outer diameter (OD), slightly less than the inner diameter (ID, 1.02 mm) of commercially available 18 Ga PICC catheters. PTFE liners, similar to those used herein, are also used for the manufacturing of coronary guide catheters^29^ and are available from fluoropolymer tubing manufacturers around the world. Outer surface of these commercially available PTFE liners is chemically etched (as supplied) for stronger adherence to the thermoplastic polyurethane. To obtain the three-layer structure shown in Fig. 1, a stainless-steel mandrel was used as the guide mandrel and inserted inside the PTFE liner. Subsequently the PTFE lined mandrel was inserted into a commercially available 18 Ga, 4Fr single lumen PICC. Finally, a commercially available 50 ± 12 micron thick, heat shrinkable FEP (FEP-HS) outer cover was sealed over the entire assembly, to construct the three-layer PTFE-TPU-FEP structure. The 1.52 mm expanded inner diameter of the FEP-HS outer cover allowed us to conveniently slide it over the PICC-PTFE lined mandrel. The entire assembly was then heated in a convection oven at 89 ºC for 15 min to soften the TPU middle layer sandwiched between the PTFE and FEP layers. The heating causes uniform redistribution of the TPU middle layer between the inner PTFE liner and outer FEP-HS cover, which simultaneously undergoes thermal shrinkage to form uniform lamination on the soft polyurethane middle layer. The inner diameter of the heat shrunk FEP cover measures 1.24 mm, closely matching the 1.33 mm outer diameter of the 4Fr PICC catheter, allowing the FEP-HS to tightly fit onto the middle layer constructed by the 4Fr PICC line. Finally, the assembly was allowed to cool down to room temperature and the stainless-steel mandrel guide was removed to obtain the three-layer structure shown in Fig. 1, where both inner and outer surfaces of the PICC line were laminated with perfluoropolymer layers. The resulting PICC catheter showed mechanical flexibility comparable to a TPU PICC (video link S2, supporting information).

### Surface analysis

**Figure 2:**
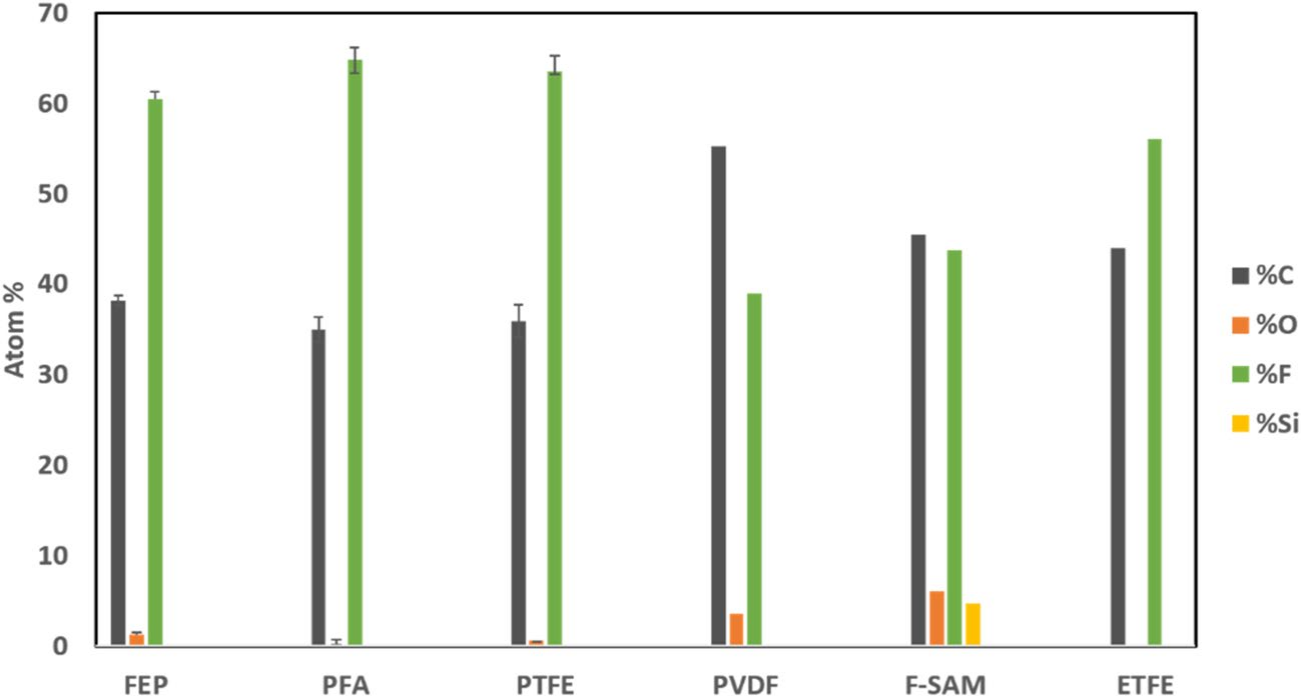
Elemental composition of various pure fluoropolymer (FEP, PFA, PTFE, PVDF, ETFE) surfaces and F-SAM coated PICC.

X-ray photoelectron spectroscopic (XPS) analysis was performed to determine the elemental composition of pure PTFE, FEP, PFA and PVDF surfaces as well as PICC line surfaces modified either by lamination with fluoropolymer (FEP & PTFE) layers or by F-Silane grafting (F-SAM/PICC). Elemental composition of pure fluoropolymer (FEP, PTFE, PFA, ETFE and PVDF) surfaces showed 61, 64, 65, 55 and 39 at.% F, respectively, which closely matched the molecular formula of those fluoropolymers, within the accuracy range (10%) of XPS analysis^30^. The results are summarized in Fig. 2. Elemental compositions of fluoropolymer (FEP and PTFE) laminated PICC line surfaces were consistent with the elemental composition of the pure fluoropolymers, which showed significantly high (> 60 atom%) fluorine compared to the elemental concentration (44 at.%) of fluorine on the F-SAM deposited PICC line surface.

### Comparing in vitro thrombogenicity of FILP PICC, LP/F-SAM PICC with commercially available BARD PICC and BioFlo PICC

**Figure 3:**
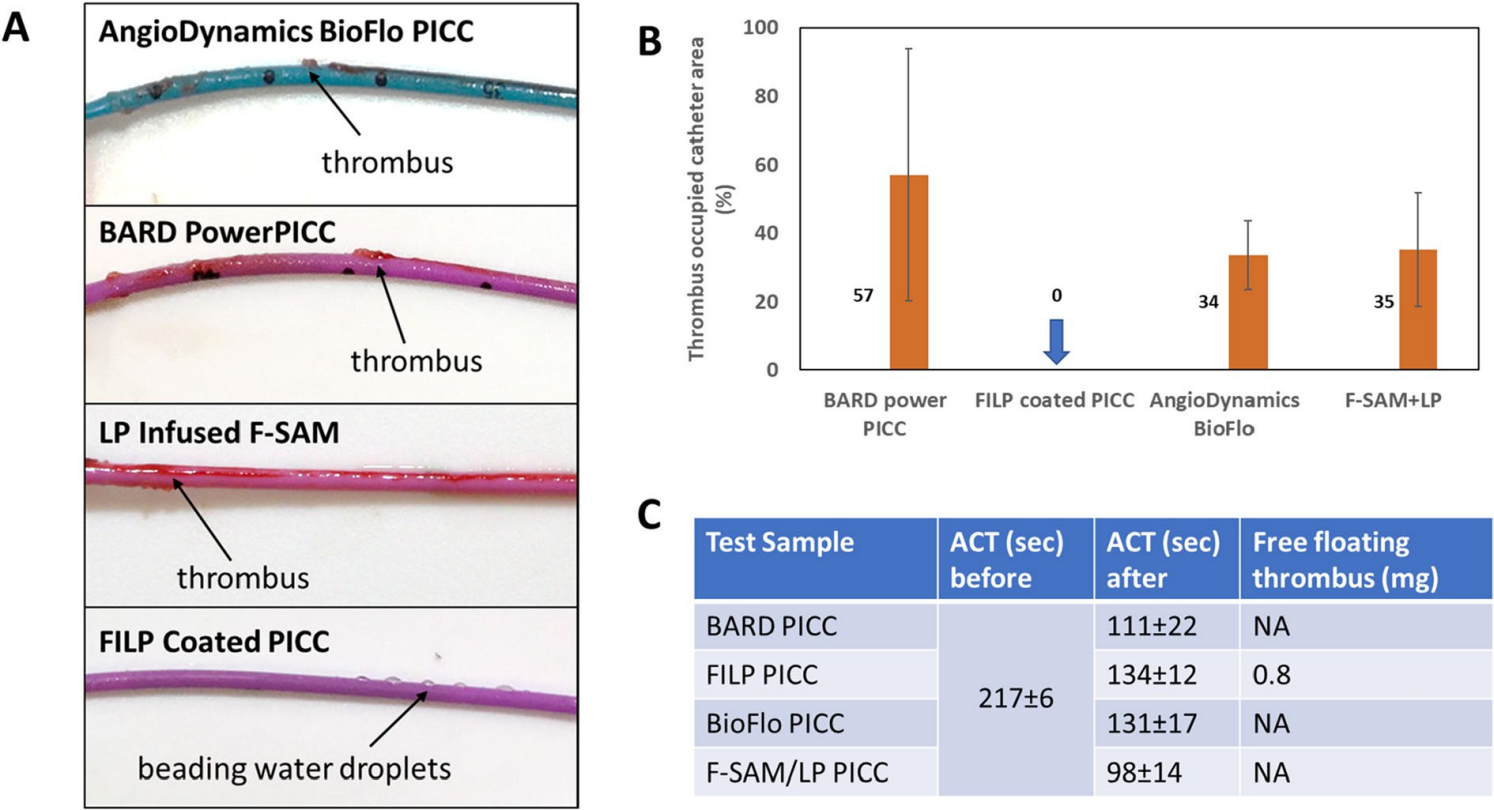
Comparative picture (**A**) of thrombus covered BioFlo PICC, BARD PICC, LP/F-SAM PICC and FILP PICC after their exposure to 500 mL/min flow of sheep blood (heparin 1 IU /mL) for 4 h. Percentage of thrombus deposited area (**B**) on all four test samples. Activated clotting time and the quantified free floating clot at the end of the thrombogenicity test suggest no significant blood clotting happened during the 4 h period of blood circulation on FILP coated PICC.

In a clinical setting, implanted PICC lines are typically exposed to a 1–10 cm/s intravenous blood flow velocity^31^. Following a standardized test protocol^32^, thrombogenicity of surface coated PICCs, namely LP/F-SAM PICC and FILP PICC was compared with two commercially available PICC lines: BARD PICC and BioFlo PICC under physiological flow conditions. For this test each PICC line was exposed to heparin stabilized (1 IU/mL) fresh ovine blood flow at 500 mL/min, similar to jugular blood flow rate^33^. Sheep blood used for this study was collected from 1–4 years old, healthy donor sheep of mixed breed, ranging 70–146 kg in weight. All donors were free from any anticoagulant medication e.g. aspirin, ibuprofen, acetaminophen, heparin or coumadin for two weeks before the blood was drawn for the thrombogenicity test. No blood was drawn from the donors during this two-week period. Porcine heparin was added during the venipuncture and collection process to yield a final concentration of 1 IU/mL. The heparin concentration used is comparable with other in vitro thrombogenicity study models published before^34^. The minimally heparinized fresh ovine blood was then analyzed for activated clotting time (ACT) which is within the therapeutic range (240–180 s) when it began circulating through a closed loop created with a 140 cm long blood perfusion tube. All catheter samples were inserted into the blood containing loop towards the direction of blood flow, using a peel away introducer and needle. This insertion procedure is clinically relevant. All PICC samples were collected and rinsed with saline at the end of the four-hour period and used for visual assessment to quantify the relative percentage of thrombus formation on the catheter surface. Since all catheter samples tested are identical cylinders with comparable diameter throughout the length, the total coverage of an identified area was calculated by taking the length of the thrombus formed multiplied by a visual estimate of the percentage of the circumference of the study article covered within the length. These segments were summed for the entire catheter, and this area was divided by the total implanted length and converted to a percentage. The thrombogenicity assessment thus obtained, as shown in Fig. 3, consistently established the absolute thromboresistance of FILP coated PICC with a 0% thrombus occupied catheter area, in all nine replicates analyzed over three repeated trials. Whereas both BARD PICC and BioFlo PICC had 57% and 34% thrombus covered surfaces, respectively. The LP infused F-SAM deposited PICC surface also showed a 35% thrombus occupied area which reflects its poor durability on the outer surface of the catheter. Digital images of a FILP coated catheter showed high contact angles of water droplets which indicates that no blood component was deposited on the catheter surface, even under the high blood flow conditions used for this test.

### Resistance to CLABSI causing bacterial colonization

**Figure 4:**
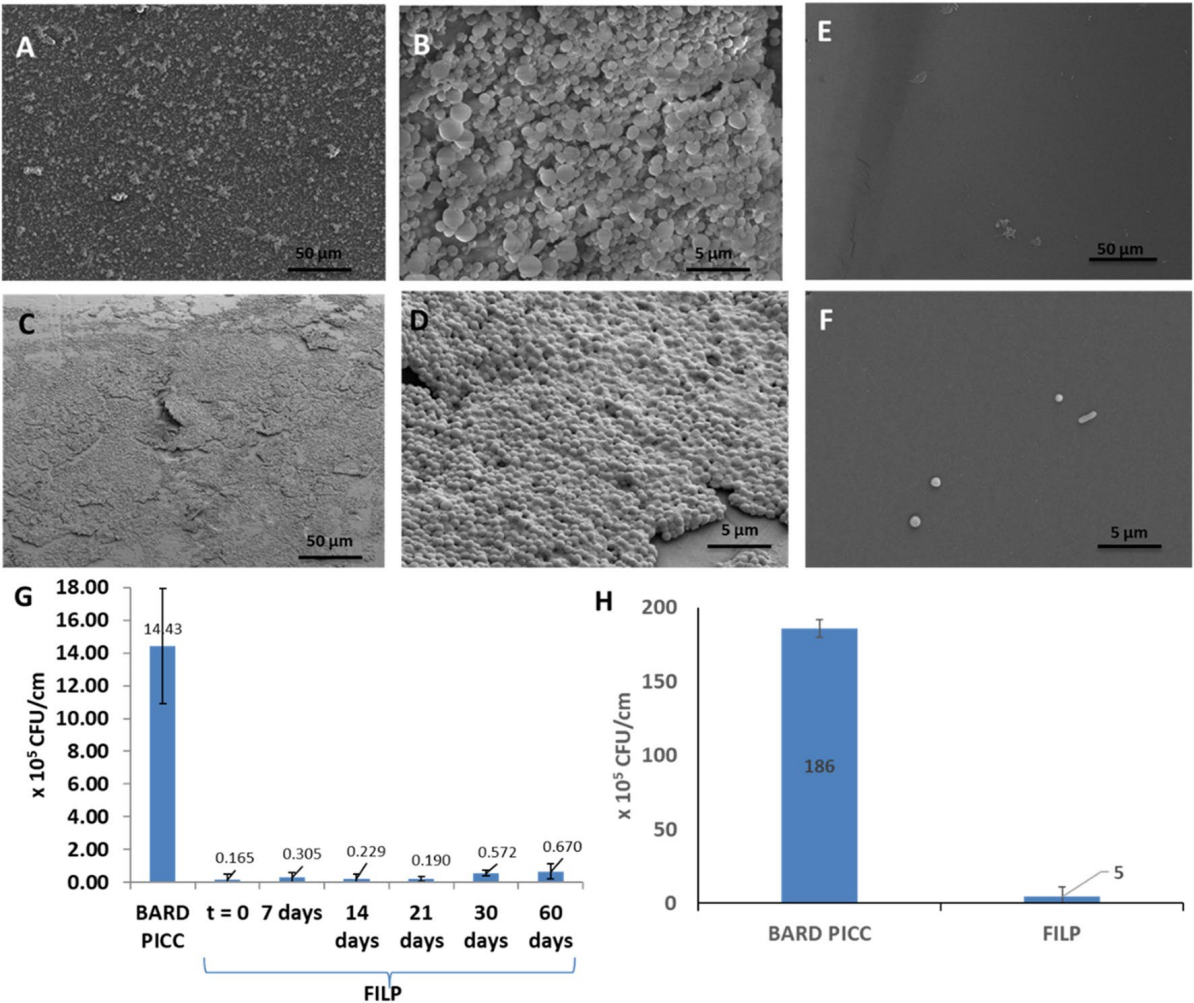
Biofilm formation on BARD PICC and FILP PICC. (**A**, **B**) *S. epi* biofilm on BARD PICC. (**C**, **D**) *S. aureus* biofilm on BARD PICC. (**E**) *S. epi* single pathogen on FILP PICC. (**F**) *S. aureus* single pathogen on FILP. A comparison of quantified *S. epi* (**G**), *S. aureus* (**H**) biofilms in terms of viable cell count per cm (CFU/cm) of BARD PICC and FILP PICC. FILP PICC samples were isolated at various time points after exposing to physiological flow and the subsequent incubation with *S. epi* pathogen.

Efficacy of FILP PICCs in resisting bacteria colonization was compared with BARD PICCs by exposing both catheter samples to cell culture media containing 0.5% glucose and either *S. epi* or *S. aureus*, both bacteria known to cause CLABSI. After 48 h of incubation at 37 °C, the *S. epi* biofilm adhered to the catheter surface was dislodged by sonication and a viable cell count assay was done on the dislodged biofilm by the quantitative culture of bacterial species using tryptic soy ager plates. The *S. aureus* biofilm was allowed to grow for 96 h at room temperature, under 1 cm/sec flow of the cell culture media and then isolated and quantified following the same protocol. The bacterial colonization, measured as the colony forming unit (CFU) observed per centimeter length of each catheter tube are shown in Fig. 4G,H. Freshly coated FILP PICCs showed 87-fold reduction of *S. epi* biofouling and 37-fold reduction of *S. aureus* biofouling in comparison with BARD PICC. Both are statistically significant (*p* < 0.00001) reductions. SEM images of PICC after exposure to bacteria solution clearly show the presence of a bacterial biofilm on the BARD PICC whereas only discrete bacterial cells, instead of a compact biofilm, was observed on FILP PICC lines, as shown in Fig. 4A–F.

For its intended use, the FILP coating needs to continuously resist biofilm formation and blood stream infections at least for the first month, when bacteria from patient’s cutaneous microflora and care giver’s hands are believed to travel through the catheter wall, hubs and internal routes, leading to hematogenous dissemination and bacteremia^35,36^. To test the durability of anti-biofouling property of FILP PICCs under physiological flow conditions^37^, FILP PICCs were inserted into a LivaNova perfusion pack (0.95 cm ID) tubing loop, previously filled with phosphate buffer saline (PBS) which was circulated to mimic a 9.5 cm/s venous flow. Catheter samples were collected at 7, 14, 21, 30, and 60-day time points and further tested as follows. Uncoated BARD PICC as positive control, freshly coated FILP PICC as negative control and the catheter samples, harvested at various time points indicated above were challenged with *S. epi* in nutrient broth at 37 °C. After 48 h of incubation of the catheter samples in the bacterial culture broth, any *S. epi* biofilm formed was dislodged and simultaneously quantified by quantitative culture of bacterial species on tryptic soy ager plates. The durability of FILP coating, shown in Fig. 4G, was evaluated in terms of antibiofouling effectiveness of FILP PICC, which is quantified as biofilm formation per centimeter length of the catheter. FILP PICC showed significant (*p* < 0.00001) reduction of *S. epi* colonization throughout the period of 60 days when compared with BARD PICC. During the first 21 days, under physiological flow, FILP PICC did not show any significant difference from freshly coated FILP PICC. Although a statistically significant reduction of antibiofouling property was observed at the 30 (*p* = 0.0027) and 60-day (*p* = 0.00618) time points, FILP PICC still showed significant (*p* > 0.00001) improvement in resisting pathogen colonization throughout the entire 60 day period, when compared to BARD PICC. The perfluorotributylamine used as the LP layer of the FILP coating does not have any cytotoxic effect on pathogens, rather it resists bacterial colonization due to the slippery character of a molecularly smooth surface^22^. Since the slippery omniphobic surface of FILP coatings resists pathogen attachment we anticipate that it will resist CLABSI pathogens from traveling through the catheter surface into the blood stream and will also resist biofilm formation on indwelling catheter surfaces. Bacteria colonies in a mature biofilm eventually develop resistance against systemic antibiotics^6,13,38^ leading to significant complications in 400,000 cancer patients suffering from PICC associated CLABSI every year^39^.

## Discussion and conclusion

**Figure 5:**
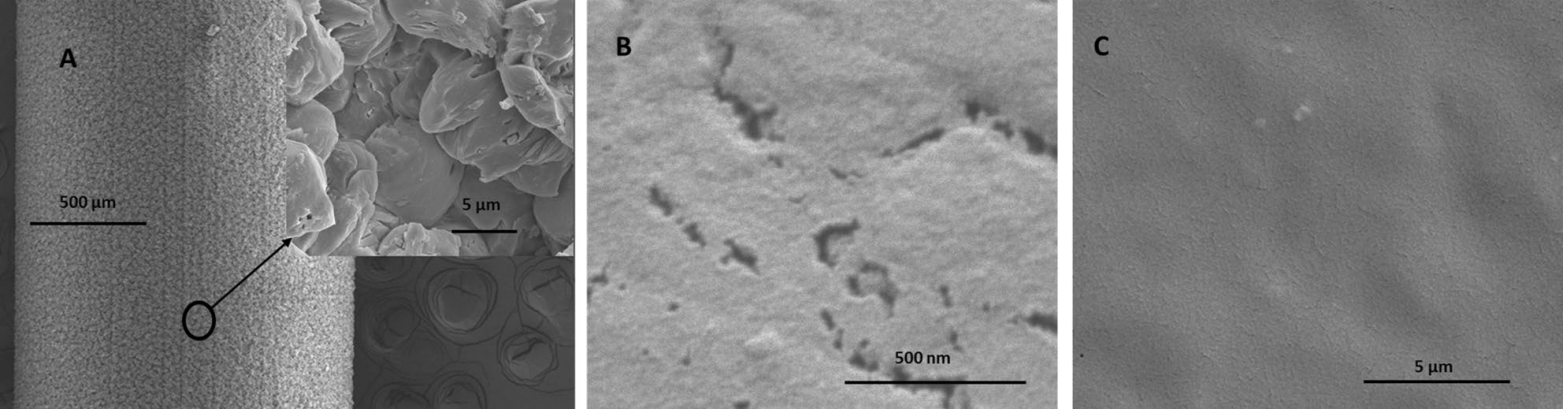
Comparison of surface roughness resulting from three different coating processes: F-SAM deposition on PICC under solution phase condition (**A**), CVD condition (**C**). Surface roughness (**B**) of FEP laminated PICC surface.

Thrombogenicity and infections are two grave problems associated with medical implants dwelling in the blood stream. Liquid perfluorocarbon (LP) infused F-SAM coated medical device surfaces have been widely studied^23,24^, because of their ability to resist both infection and thrombosis. Our initial attempt of coating commercially available PICC lines by a solution phase F-SAM deposition process yielded a rough surface, as evident from the SEM image shown in Fig. 5. The surface roughness most likely results from the degradation of the polyurethane by evolution of solution phase HCl during the F-silane condensation step. The alternative CVD approach produced a smooth surface, as evident by SEM imaging, and the uniformity of F-SAM deposition was confirmed by XPS analysis at multiple locations on the catheter which showed 44 at.% fluorine. The liquid perfluorocarbon used in the slippery omniphobic coating is believed to be adhered to the fluorinated medical device surface by Van-der Waals force^40^. We compared the ability of different fluoropolymers to retain the LP layer against shear force by a simple dipping/flow experiment, paired with slide angle measurements. We discovered that the perfluoropolymers PTFE, FEP and PFA, which do not have any hydrocarbon residue in their molecular structure, can retain an LP for a longer time than PVDF and ETFE, which have hydrocarbon moiety in their polymer backbones. In this context, it is noteworthy that ETFE fails to retain LP on its surface although it has > 55 at.% fluorine which is higher than what was ever reported with F-SAM deposition approach.

To the best of our knowledge, the current study describes a smooth fluoropolymer lamination approach, for the first-time, to obtain an omniphobic FILP coating on medical device surface. High resolution SEM imaging (500 nm scale) of the fluoropolymer laminated on the PICC surface, shown in Fig. 5, confirmed that a smooth fluoropolymer surface as comparable to previous studies^41^ was maintained after the catheter manufacturing process. The effect of fluoropolymer roughness on the durability of liquid perfluorocarbon layer when exposed to high shear conditions is currently under investigation.

The fluoropolymer lamination described in this FILP coating process can be completed in < 20 min and could be easily translated into the standard catheter extrusion process with minor process modifications, where TPU will be directly extruded over the pre manufactured PTFE liner, and the heat shrinkable FEP outer cover will be applied at the end of the extrusion line. The F-SAM deposition approach is generally lengthy (> 20 h) and the potential leaching of surface grafted F-SAM over the period of time is yet to be established by in vivo biocompatibility studies. The FILP coating replaces F-SAM with the fluoropolymers PTFE and FEP which are already being used in various FDA approved medical devices. The LP such as perfluorodecalin, has found use in opthalmic surgery, and also as liquid ventilation^42^ for delivering oxygen as well as blood substitute^43^. The lamination approach followed herein to manufacture FILP coating produces a more uniformly fluorinated surface with a higher atomic concentration of fluorine (> 60 at.% fluorine) than an F-SAM deposited surface (44 at.% fluorine). The uniformity of the latter is dependent on the reactivity of the medical device surface towards plasma activation and the subsequent F-silane grafting step. Uniformity of the high atomic concentration of fluorine is the key factor to enhance the Van-der Waal’s affinity of the liquid perfluorocarbon towards the fluorinated medical device surface. These make FILP coatings more time efficient, uniform, and safe than an LP infused F-SAM coating.

Slippery omniphobic coatings do not activate coagulation cascades triggered by contact pathways^24^ which involve the activation of coagulation factors XI and XII. Our results also show that FILP coatings results in higher thromboresistance, a key property of omniphobic slippery coatings, than LP infused F-SAM coatings on PICC surfaces. When the thromboresistance of an LP infused F-SAM coating on the PICC catheter was measured by exposing it to 500 mL/min whole blood flow for 4 h, it showed marginal improvement over a commercially available BARD PICC and no significant improvement over a BioFlo PICC which uses a fluoropolymer additive^20^ to enhance the thromboresistance of TPU PICCs. In the same experiment, FILP PICC consistently showed no thrombus deposition indicating highest thromboresistance the test can permit. Our thrombogenicity test uses a very low (1 IU/mL) heparin concentration as others reported in the literature^34,44^ and direct visual assessment of thrombus formation which is currently accepted by the ISO-10993-4 guideline followed during an in vivo hemocompatibility test on nonanticoagulated venous implant (NAVI) model. Also this in vitro model has been compared^32^ with the in vivo NAVI model and is currently offered by American Preclinical Services following GLP standards.

Another key feature of the slippery omniphobic coating is its ability to physically resist bacterial adhesion and colonization and thus resist biofilm formation which is known to cause significant morbidity and suffering. We have also shown that FILP PICCs resist colonization of *S. epi* by 99% and *S. aureus* by 95%, both of which are known to be responsible for CLABSI. Furthermore, the FILP coating continues to maintain its anti-biofouling property for over 60 days, a timeline which is well beyond the initial 14–21 days after catheter implantation, when the extraluminal pathway is known as the primary source for CLABSI^36^. The ability of a FILP coated CVL to resist fibrin sheath formation and biofouling will be evaluated in long term in vivo studies as part of our ongoing research and development program, with the goal of finding an antibiotic free solution for CLABSI. The FILP coating technology developed and demonstrated on PICC catheters will potentially improve antithrombogenicity and infection resistance of other indwelling medical implants and reduce the usage of antibiotics and systemic anticoagulants and associated complications. Application of FILP coating technology is not only limited to medical devices but will also significantly improve antibiofouling property of any commercially available material surface, fluoropolymer-coated to decrease biofouling and material adhesion. This coating process is also directly applicable to food processing and packaging industry where PFA and FEP lined polyvinyl chloride (PVC) tubes and containers are extensively used. Our data strongly suggests that FILP coating will reduce the risk of biocontamination and provide a self-cleaning property due to its slippery surface. Additionally, this coating will provide self-cleaning property to any fluoropolymer lined chemical transfer lines used in chemical industry.

## Materials and methods

### Materials

Extruded PTFE (outside etched, inside natural) and heat shrinkable FEP liners were purchased from Zeus. Perfluorodecalin was purchased from exfluor research. Perfluorotributylamine and perfluorotripentylamine were purchased from Santa Cruz Biotechnology Inc. Trichloro(1H, 1H, 2H, 2H perfluorooctyl) silane and other common chemicals were purchased from Gelest. Whole blood was purchased from Lampire. Human plasmin was purchased from Affinity Biological Inc. Bacterial strains, *S. epi* (ATCC 14,990) and *S. aureus* (ATCC 25,923) were purchased from ATCC. Cell culture broth and supplies were purchased from Carolina Biopharmaceuticals. Single lumen BARD PICC were generously donated by Dr. Gidon Ofek from BARD. PTFE and FEP liners were custom manufactured at Zeus Inc. All fluoropolymer films and tubing were purchased from McMaster Carr. Pebax was purchased from Chamfr. Blood perfusion tubing was purchased from LivaNova. Peel away introducers were purchased from MILA.

Ethical approval and protocol for sheep blood collection: the use of all the animals in this study was with the review and approval of the American Preclinical Services Institutional Animal Care and Use Committee (IACUC). American Preclinical Services is licensed by the United States department of agriculture (USDA) and is AAALAC accredited. Veterinary medical care program is patterned after the principles outlined in the NIH Guide for the Care and Use of Laboratory Animals. Veterinary care is executed by the animal facility staff following APS standard operating procedures (SOPs) that address issues such as sanitation, quarantine, necropsy and euthanasia procedures, monitoring physical environment, disease control procedures and surgical/interventional procedures. Fresh blood was collected from healthy donor sheep. Each sheep was free from aspirin, acetaminophen, heparin, Coumadin, and other thinning medications for a minimum of 7 days prior to draw. Subcutaneous lidocaine was administered over the jugular vein then large bore venipunctures kits (14–16 gauge catheters) were used to access the vessel. Gravity flow was used to draw blood from the animal into a minimally pre-heparinized collection bag. A scale was used to measure the approximate volume of blood. The volume of blood drawn over did not exceed 0.8% of the animals total bodyweight, in accordance with APS IACUC approved blood draw protocol. Immediately following collection, intravenous isotonic fluid was administered at a volume equal to amount of blood drawn. Each animal was then given a minimum 14 day rest period in between blood draws to allow for adequate recovery. The minimally heparinized blood was then transported to the in vitro lab and placed on a nutating rocker in an incubator heated at 37 °C until the ACT is measured and confirmed to be within 150 to 250 s. Blood was then added to each of the loops within 60 min of the completion of the draw.

### Preparation of fluoropolymer laminated three layer PICC (PTFE–TPU–FEP)

On a 78 cm long stainless steel wire mandrel (0.64 mm diameter), a 56 cm long PTFE liner (0.81 mm ID, 0.89 mm OD) was inserted followed by single lumen, 4 Fr BARD PICC and 56 cm long, heat shrinkable FEP cover (1.52 mm minimum expanded ID, 0.051 mm wall, 1.24 mm maximum recovered ID, and 1.34 mm maximum recovered OD). The final assembly was placed in a convection oven set at 89 °C for 15 min. After this heating step the assembly was allowed to cool down to room temperature and the stainless-steel wire was pulled out to produce the three layer fluoropolymer modified PICC (PTFE–TPU–FEP).

### Applying liquid perfluorocarbon on fluoropolymer films and coated PICC surface

Each LP layer (perfluorodecalin, perfluorotributylamine and perfluorotripentylamine) was applied using a paint brush on all fluoropolymer films (PTFE, FEP, PFA, ETFE and PVDF), and F-SAM modified Pebax films for the dipping experiment and for the contact angle and slide angle measurements. Catheter samples and fluoropolymer tubing were submerged into LP for 3 s to thoroughly lubricate the surface. All LP coated test articles were submerged vertically into deionized water for one second to remove excess LP before employing them in any experiment.

### Contact angle and slide angle measurements

All polymer films to be tested were secured on glass slide with paper clips, to measure the contact and sliding angles, using 5 µL droplets of deionized water. The water sessile drop technique was used at room temperature to measure all water contact angles using Theta Optical Tensiometer 200 which was calibrated prior to each measurement. Sliding angles were measured using a custom-made test setup utilizing a tilt table. Five drops, each of 5 µL volume were placed on the test surface using a multichannel micropipette. The glass slide surface was gradually tilted and sliding angle was recorded at the minimum tiling angle when more than three droplets started moving. All contact angles and sliding angle measurements were repeated three times at different areas of the test slide.

### Thromboresistance test with minimally heparinized whole blood

140 cm of blood perfusion tubing (Livanova perfusion pack, 3/8 in ID), was used to create a loop for each individual catheter type to be tested. Blood was drawn via gravity flow from a healthy sheep into a collection bag pre-loaded with heparin at approximately 1 IU/mL heparin to blood ratio. The ACT of the blood was measured and confirmed to be within 150 to 250 s prior to testing. Each loop was first filled partially with sheep whole blood, then each catheter was inserted through 6Fr × 3.75 cm peel away introducer into the partially filled loop in the direction of blood flow. Up to three identical (10 cm) replicates of each catheter were inserted through the tubing wall into each loop, leaving 2 cm outside of the tubing to hold each replicate in place for testing. A minimum of 5 cm distance was kept between the distal tip of one catheter replicate and the insertion site of the next catheter replicate. Insertion sites were sealed with parafilm then the loops were filled completely and closed after all air was removed from the system. The blood was continuously circulated throughout the loop using a peristaltic pump set to 500 mL/min for 4 h ± 30 min and heated at 37 ± 2 °C using a water jacket pad and Lab Armor beads. After the exposure time each loop was drained of blood and splayed open to remove the catheters. Each catheter was rinsed gently with saline to remove residual blood, then evaluated for adherent thrombus. Percent thrombus formation was calculated using a calibrated ruler to measure the length of each thrombus segment. This length was then multiplied by the percent of the circumference that is covered by the thrombus segment and divided by the total length of the device to determine the percent coverage of each individual thrombus formation. Multiple thrombus formations were added together to determine the total percent thrombus coverage of each catheter.

### Evaluation of anti-biofouling property and its durability under physiological flow conditions

Control BARD PICCs and fluoropolymer laminated PICC catheters were sterilized by 12 h exposure of ethylene oxide. LP was filter sterilized using a nylon sterile filter having a 0.2 µm porosity.

In a biosafety cabinet fitted with a HEPA filter, FILP PICC was prepared right before the experiment by submerging the sterile fluoropolymer laminated PICC into the filter sterilized LP.

#### S. aureus biofouling comparing BARD PICCs and FILP PICCs under flow conditions

Inside a sterile biosafety cabinet fitted with a HEPA filter, TSB containing 0.5% glucose and 3% NaCl was filled into a 91.5 cm long Livanova blood perfusion tube (perfusion pack ID 0.95 cm) assembled together with barb connectors and previously sterilized by ethylene oxide exposure for 12 h. A series of 10 cm long FILP PICC catheters in triplicate was dangled inside the tubing towards the direction of flow using a Dyneema suture secured at the barb connection, where the tubing was connected to an identical setup containing 10 cm long BARD PICC catheters. These two lengths of tubing were closed into a loop by connecting to a 38 cm length of Masterflex 06434-17 tubing (chosen for compatibility with the peristaltic pump head). The assembled loop was inoculated with 1.57 mL (1% of the total volume of cell culture media inside the loop) of *S. aureus* broth and all air bubbles were removed. The cell culture media was circulated through the loop with a peristaltic pump at 18 mL/min for 48 h after which visible biofilm was observed on the BARD PICC. After this incubation period all test catheters were isolated from the flow loop, cut into 2 cm segments, and the biofilm from each segment was dislodged into 2 mL PBS in a 2 mL microcentrifuge tube by 15 min of sonication in a Branson M1800 bath sonicator followed by 3 min of vortexing. The suspended biofilm was quantified by quantitative bacterial culture in tryptic soy agar.

#### Physiological flow setup

Inside a sterile biosafety cabinet fitted with a HEPA filter, sterile phosphate buffered saline (PBS) was filled into a 48 cm length of PVC tubing (Thermo Scientific 180 PVC, 1/4″ ID, 3/8″ OD) assembled together with barb connectors and previously sterilized by ethylene oxide exposure for 12 h. Three 13 cm long FILP PICC catheters were dangled into the loop using a Dyneema suture secured at the barb connection. The PVC tubing was terminated with 1/4″ barb luer connections, then formed into a closed loop by connection to a 25 cm length of Masterflex 06434-17 tubing, also terminated with luer connections. The completed loop was placed on a Fisher Scientific peristaltic pump 50 rpm and aged to test durability of the FILP coating against 180 mL/min flow. Four additional loops were prepared in the same manner and placed under the same flow conditions in order to test durability of FILP coating at 7 days, 14 days, 21 days, 30 days, and 60 days.

#### Evaluation of anti-fouling durability of FILP PICC by challenging with S. epidermidis

After aging under flow conditions, the FILP catheters were removed from the flow loop and challenged, alongside BARD PICCs (positive control) and fresh FILP catheters (negative control) by *S. epidermidis*. The loop was removed from the pump and cut open lengthwise to access the FILP catheters. Care was taken to not expose the samples to air, minimizing any evaporation of the LP from the catheter. To do so, the FILP catheter samples were cut into 26 mm lengths while still submerged in PBS, then pulled into a pipette containing 1 mL PBS. Each cut sample, along with the 1 mL of PBS, was then transferred into individual 50 mL centrifuge tubes, each containing 5 mL of nutrient broth and 0.5 mL glucose. Additionally, a 22 mm^2^ glass cover slip was placed in each centrifuge tube in order to help visualize biofilm growth. Centrifuge tubes containing positive controls (26 mm BARD PICCs) and negative controls (26 mm fresh FILP catheters), all previously sterilized with ethylene oxide exposure, were also prepared in the same manner. Each centrifuge tube was inoculated with 10 µL of *S. epidermidis* broth then incubated at 37 °C for 24 h. After 24 h of growth, each sample and coverslip were propagated into a new 50 mL centrifuge tube containing 5 mL of fresh nutrient broth and 0.5 mL glucose. 20 µL of broth from the old centrifuge tube was used to inoculate the new tube, then the tubes were incubated for another 24 h at 37 °C. After a total of 48 h of biofilm growth, each sample was removed from its centrifuge tube and rinsed by gently dipping in 2 mL of nutrient broth. Each sample was then placed in its own 2 mL microcentrifuge tube containing 1.5 mL of nutrient broth. In order to delaminate the biofilm from the samples, the microcentrifuge tubes were placed in a water bath and sonicated for sixty seconds at full amplitude and frequency with a Hielscher UP400S (400 W, 24 kHz) sonic probe. The suspended biofilm in each tube was then quantified by quantitative bacterial culture in tryptic soy agar.

### X-ray photoelectron spectroscopy

All samples were analyzed at three separate areas and the mean atom percent value was reported for each target area. Photospectroscopy (XPS) measurements were performed using a Kratos Axis Ultra. A monochromated Aluminum K-alpha X-ray source was used operating at 15 kV and 10 mA. Survey scans were performed using a pass energy of 160 eV and a step size of 1 eV, while carbon region scans were performed using a pass energy of 20 eV and a step size of 0.1 eV. A charge neutralizer was employed for all measurements, and data was calibrated to the C–C peak using a value of 284.8 eV. Recorded raw data was analyzed using instrument software and the atom percent of carbon, oxygen, fluorine and silicon were reported.

### Scanning electron microscopy

After the biofouling experiment the biofilm on catheter was fixed by incubating catheter samples for four hours into 50 mL fixative solution (10 mL 20% formaldehyde, 4 mL 20% glutaraldehyde, 5 mL 10X PBS and 31 mL deionized water). Catheter samples were removed from the fixative and rinsed with deionized water and stored at 2–8 °C until analysis. Scanning Electron Microscope (SEM) images were taken on a ThermoFisher Scientific Apreo SEM. The samples were first sputter coated on an approximately 10 nm thick gold layer, then imaged using an accelerating voltage of 2 keV.

### Statistical analysis

A one-way ANOVA was used to test the statistical difference between the colony forming units in comparative biofouling study and *p* values < 0.05 were considered statistically significant. All experiments were repeated at least three times and all data are presented as mean with ± standard deviation.

## Supplementary information

Supplementary Information.

Supplementary Video 1.

Supplementary Video 2.

## Data Availability

The data generated during the study are available from the corresponding author on reasonable request.
