# Supplementary material for: Slippery liquid infused fluoropolymer coating for central lines to reduce catheter associated clotting and infections
Source: Sci Rep. 2020 Sep 11;10:14973. doi: 10.1038/s41598-020-71711-6 (PMC7486915; doi:10.1038/s41598-020-71711-6)
Supplement: Supplementary file 1 — Supplementary Information. [file 41598_2020_71711_MOESM1_ESM.docx]

**Supporting Information**

**Slippery Liquid Infused Fluoropolymer Coating for Central Lines to Reduce Catheter Associated Clotting and Infections**

**Authors:** Saibal Bandyopadhyay*^1^, Andrew Jones^1^, Andrew McLean^1^, Matthew Sterner^1^, Carolyn Robbins^1^, Matthew Cunningham^2^, Mark Walters^3^, Kiran Doddapaneni^1^, Isaac Keitel^1^, Colin Gallagher^1^

^1^FreeFlow Medical Devices LLC, Lancaster, PA, USA

^2^American Preclinical Services LLC, Minneapolis, MN, USA

^3^Shared Material Instrumentation Facility, Duke University, Durham, NC, USA

Correspondence and request for material should be addressed to Saibal Bandyopadhyay (email: saibal@freeflowmed.com)


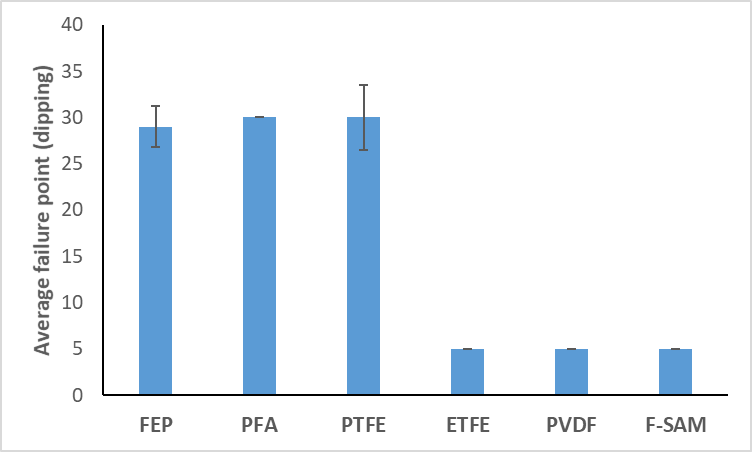


**Figure S1:** Average failure point of low sliding properties of LP coated fluoropolymer and F-SAM deposited films due to the shear stress after subsequent dipping.

**Dipping test to discover fluoropolymer which can best retain the LP layer**

All fluoropolymer films (PTFE, FEP, PFA, ETFE and PVDF), Pebax film, and an F-SAM modified Pebax film were secured side by side using paperclips on an aluminum plate (15.24 cm x 30.48 cm) then the LP layer was applied on all films except for the untreated Pebax control film. The fully assembled aluminum plate was vertically submerged consecutively into a deionized water bath 5, 10, 15, 20, 25, 30, 35 times. After every set of dippings, the aluminum plate was placed at a 5 degree tilt angle and five water drops (5 µL each) were gently placed on each test surface using a multichannel micropipette. When more than three droplets moved we concluded that the low slide property was maintained. The dipping experiment was repeated five times, each time changing the sequence of the films tested.


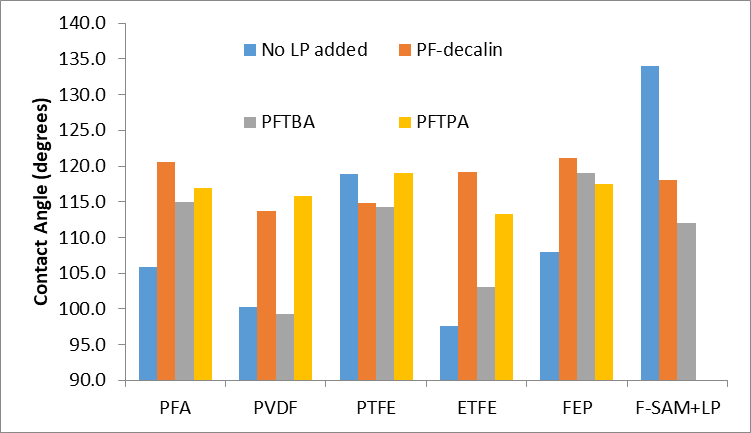


**Figure S2.** Water contact angles on fluoropolymer films lubricated with perfluorodecalin (PF-decalin), perfluorotributylamine (PFTBA), and perfluorotripentylamine (PFTPA)


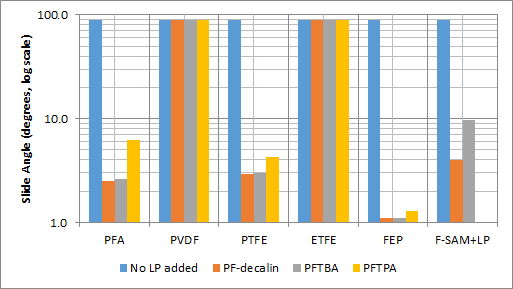


**Figure S3.** Slide angle of 5µL drops of water on fluoropolymer films lubricated with perfluorodecalin (PF-decalin), perfluorotributylamine (PFTBA), and perfluorotripentylamine (PFTPA)

**Table S1.** Water contact angles on fluoropolymer films lubricated with perfluorodecalin, perfluorotributylamine, and perfluorotripentylamine

|  | PFA | PVDF | PTFE | ETFE | FEP | F-SAM+LP |
| --- | --- | --- | --- | --- | --- | --- |
| No LP Added | 105.8 | 100.3 | 118.9 | 97.6 | 108.0 | 134.1 |
| Perfluorodecalin (PFD) | 120.6 | 113.8 | 114.8 | 119.1 | 121.1 | 117.9 |
| Perfluorotributylamine (PFTBA) | 115.0 | 99.3 | 114.3 | 103.1 | 119.0 | 111.6 |
| Perfluorotripentylamine (PFTPA) | 116.9 | 115.8 | 119.1 | 113.3 | 117.5 | N/A |

**Table S2.** Slide angle of 5µL drops of water on fluoropolymer films lubricated with perfluorodecalin, perfluorotributylamine, and perfluorotripentylamine

|  | PFA | PVDF | PTFE | ETFE | FEP | F-SAM+LP |
| --- | --- | --- | --- | --- | --- | --- |
| No LP Added | 90.0 | 90.0 | 90.0 | 90.0 | 90.0 | 90.0 |
| Perfluorodecalin (PFD) | 2.5 | 90.0 | 2.9 | 90.0 | 1.1 | 4.0 |
| Perfluorotributylamine (PFTBA) | 2.6 | 90 | 3 | 90 | 1.1 | 9.7 |
| Perfluorotripentylamine (PFTPA) | 6.2 | 90 | 4.3 | 90 | 1.3 | N/A |

**Table S3.** Surface tension and viscosities of liquid perfluorocarbons used.

| Liquid perfluorocarbon | surfce tension (mN/m) | viscosity (mm2/s) |
| --- | --- | --- |
| Perfluorodecalin | 17.6 | 2.66 |
| Perfluorotributyl amine | 16 | 2 |
| Perfluorotripentyl amine | 18 | 12 |

**Video S1.** Films with heparinized sheep blood at 5-degree slide angle.

<https://neverwet.wistia.com/medias/4rmeib6s4i>

**Video S2.** Showing PICC catheter with mechanical flexibility comparable to TPU PICC.

<https://neverwet.wistia.com/medias/1twcfihfif>
